# Post-contrast Susceptibility Weighted Imaging in Multiple Sclerosis MRI Improves the Detection of Enhancing Lesions

**DOI:** 10.1007/s00062-025-01508-5

**Published:** 2025-03-07

**Authors:** Pablo Naval-Baudin, Karen Pérez-Alfonso, Albert Castillo-Pinar, Ignacio Martínez-Zalacaín, Pablo Arroyo-Pereiro, Lucía Romero-Pinel, Nahum Calvo, Antonio Martinez-Yélamos, Mónica Cos, Sergio Martínez-Yélamos, Albert Pons-Escoda, Carles Majós

**Affiliations:** 1 Radiology Department, Hospital Universitari de Bellvitge, L’Hospitalet de Llobregat, Carrer de Feixa Llarga SN, 08907 Barcelona, Spain; 2 Institut de Diagnòstic Per La Imatge (IDI), L’Hospitalet de Llobregat, Centre Bellvige, Carrer de Feixa Llarga SN, 08907 Barcelona, Spain; 3 Departament de Ciències Clíniques, Facultat de Medicina I Ciències de La Salut, Universitat de Barcelona (UB), Carrer de Casanova 143, 08036 Barcelona, Spain; 4 Multiple Sclerosis Unit, Department of Neurology, Hospital Universitari de Bellvitge, L’Hospitalet de Llobregat, Carrer de Feixa Llarga SN, 08907 Barcelona, Spain; 5 Neurological Diseases and Neurogenetic Research Group. Bellvitge Biomedical Research Institute (IDIBELL), L’Hospitalet de Llobregat, 08907 Barcelona, Spain; 6 Translational Imaging Biomarkers Group. Bellvitge Biomedical Research Institute (IDIBELL), L’Hospitalet de Llobregat, 08907 Barcelona, Spain; 7 Neuroncology Unit. Bellvitge Biomedical Research Institute (IDIBELL), L’Hospitalet de Llobregat, 08907 Barcelona, Spain

**Keywords:** Multiple sclerosis, Susceptibility-weighted imaging, T1-weighted imaging, Gadolinium-based contrast agent, Magnetic resonance imaging

## Abstract

**Objectives:**

MRI is essential for monitoring multiple sclerosis (MS). Contrast-enhanced T1-weighted imaging (T1WI+C) detects active inflammatory lesions indicating blood-brain barrier breakdown and is relevant for disease monitoring and treatment optimization. Susceptibility-weighted imaging (SWI) may be included in the imaging protocol for detecting MS-specific features, such as the presence of central veins or paramagnetic rim lesions. However, post-contrast SWI (SWI+C) has an inherent “T1 shine-through effect” that enables the visualization of contrast-enhancing lesions. This study evaluates whether SWI+C in addition to standard T1WI+C improves the detection of enhancing lesions in patients with MS.

**Materials and Methods:**

The images of 310 patients with MS who underwent a standardized MRI protocol including T1WI+C and SWI+C using a 3T scanner were retrospectively reviewed. A neuroradiologist and radiology resident independently evaluated the images obtained on T1WI+C alone and T1WI+C plus SWI+C. The efficacy of T1WI+C alone was compared with that of T1WI+C plus SWI+C for detecting active enhancing MS lesions.

**Results:**

The neuroradiologist detected 117 lesions on T1WI+C and 123 lesions on T1WI+C plus SWI+C. The resident detected 108 lesions on T1WI+C and 121 lesions on T1WI+C plus SWI+C. The interobserver agreement improved from 0.981 to 1.00 with the addition of SWI+C.

**Conclusion:**

Adding SWI+C to standard T1WI+C consistently enhances the detection of active enhancing inflammatory MS lesions and the interobserver agreement. If standardized, this combined approach may allow for earlier detection of disease activity and improve monitoring of MS progression, potentially leading to optimized treatment decisions and improved patient outcomes.

## Introduction

Multiple sclerosis (MS) is a chronic immune-mediated disease characterized by demyelination and axonal loss in the central nervous system [1]. MRI is an essential tool for diagnosing and monitoring MS lesions. Performing MRI with intravenous gadolinium-based contrast materials further allows the identification of active enhancing lesions, which represent areas of blood-brain barrier breakdown and acute inflammation. These active enhancing lesions are key markers of dissemination in time for diagnosing MS [2] and evaluating disease activity in both clinical practice and trials [3, 4]. For this reason, international guidelines recommend using contrast-enhanced T1-weighted sequences for diagnosing and monitoring MS in different scenarios [5, 6]. Alterations in diffusion imaging, have also been proposed as possible markers of acute inflammation [7, 8], but gadolinium enhancement remains the gold standard for detecting acute inflammatory activity in MS [5, 9].

Susceptibility-weighted imaging (SWI) is a technique that employs the magnetic susceptibility of tissues to create images. It can detect iron deposition, calcifications, and hemorrhage with greater sensitivity than conventional T2-weighted MRI [10]. In the context of MS, SWI can detect clinically useful signs, such as the presence of central veins or the iron-rim sign, which can have diagnostic or prognostic value [11, 12]. Current MS neuroimaging guidelines present SWI as an optional sequence in diagnostic MRI but not for disease follow-up [5, 6]. This is motivated by the fact that the most well-established clinical use of SWI in patients with MS is the detection of a central vein, which has diagnostic but not disease-monitoring use [13]. However, more recently, studies have shown that detecting iron-rim lesions might have both diagnostic and prognostic value, making SWI potentially useful for disease monitoring [12].

Interestingly, although SWI is primarily sensitive to T2* susceptibility effects, it also retains a component of T1 weighting when the flip angle is near or above the Ernst angle [14]. This results in a ‘T1 shine-through’ phenomenon, wherein tissues or lesions with inherently shortened T1 relaxation times (for example, due to gadolinium accumulation or the presence of subacute hemorrhage) appear hyperintense on SWI. Far from being merely an artifact, this phenomenon is predictable and can be leveraged clinically to highlight subtle gadolinium enhancement in active MS lesions [14, 15]. Do Amaral and colleagues explored this effect among patients with MS and demonstrated that post-contrast SWI (SWI+C) was more sensitive to detecting active enhancing lesions than conventional contrast-enhanced T1-weighted imaging (T1WI+C) [16]. These previous works have thus shown that SWI alone may have diagnostic and prognostic utility in MS and that the T1 shine-through effect can increase sensitivity to gadolinium enhancement. However, these studies involved smaller patient cohorts and did not simulate clinical practice by integrating SWI+C alongside T1WI+C reading.

Considering the abovementioned developments, this study aimed to evaluate the utility of adding SWI+C to the standard MRI with contrast MS imaging protocol alongside T1WI+C for improved detection of active enhancing MS lesions. We hypothesized that combining these imaging techniques would offer a more accurate assessment of disease activity.

## Materials and Methods

### Ethical Considerations

Our center’s Research Ethics Committee approved this study for publication and waived the requirement for informed consent owing to its retrospective, noninterventional nature as well as the confidentiality protection measures adopted.

### Study Design

The study was conducted at a Spanish public university hospital that is part of the national reference network for patients with MS. At our center, MRI ordered for the diagnosis or follow-up of MS is performed following a unified MRI protocol using a Philips Achieva 3T scanner. In this retrospective observational study, all patients sequentially included in this scanner’s registry from August to November 2019 were evaluated. MRI studies ordered by the center’s MS unit and with an imaging protocol including at least post-contrast 3D-T1-TFE, FLAIR, and SWI were included. Conversely, MRI studies yielding a low image quality based on visual assessment were excluded.

### Imaging Protocol

All MRI studies were performed using the same Philips Achieva 3T scanner, with either a 32-channel head coil or a 16-channel head and neck coil and a unified imaging protocol. The sequences analyzed included FLAIR, T1WI+C (3D-T1-TFE), and SWI (Philips SWI with phase enhancement [SWIp]). The sequence parameters were as follows: 3D-T1-TFE: TE: 4.9 ms, TR: 10 ms, flip angle: 8°, inversion-time 1034 ms, matrix: 512 × 512, slice thickness: 1 mm, in-plane resolution dimension: 0.46 × 0.46 mm; 3D FLAIR: TE: 309 ms, TR: 5500 ms, flip angle: 40°, matrix: 512 × 512, slice thickness: 1.1 mm, in-plane resolution: 0.49 × 0.49 mm; SWIp: TE double echo: 7.2 and 13.4 ms, TR: 31 ms, flip angle: 17°, matrix: 768 × 768, slice thickness: 2.4 mm, in-plane resolution: 0.3 × 0.3 mm. The intravenous contrast (gadobutrol: 1 mmol/mL, 0.1 mmol/kg) was administered with a delay of at least 5 min before contrast-enhanced SWIp. Contrast-enhanced 3D-T1-TFE images were acquired just after the 2‑min SWIp.

In our center, SWIp was acquired after contrast administration in order to improve the conspicuity of central vein sign detection [17].

### Image Review

The acquired images were locally exported from the hospital’s Picture Archiving and Communication System to an independent workstation. Only the FLAIR, post-contrast 3D-T1-TFE, and SWI+C images were exported. The images were then anonymized before assessment by two independent readers. Reader 1: (a neuroradiologist with 3 years of specialist experience) and Reader 2 (a 2nd-year radiology resident) independently evaluated the anonymized images. First, they assessed T1WI+C in conjunction with FLAIR to identify any enhancing lesions. Next, they reassessed the cases with the addition of SWI+C. Any newly identified lesion on SWI+C was cross-checked on T1WI+C to see if subtle enhancement had been overlooked.

For analysis, we recorded:

- Lesion count: the total number of enhancing lesions identified in each reading strategy.
- Binary presence of lesions: whether ≥1 enhancing lesion was present (yes/no).

### Data Analysis

The number of enhancing lesions detected per image review method per reader and the number of patients with any number of enhancing lesions per image review method per reader were assessed. To evaluate the interobserver agreement between the two readers for each strategy, we used two metrics: 1) lesion count and 2) presence of binary lesions. The lesion count was utilized to assess the agreement based on the number of enhancing lesions detected per patient.

Statistical significance was evaluated with the paired Wilcoxon signed-rank test for lesion counts and the McNemar test for binary presence of lesions. Interobserver agreement was assessed by Pearson’s correlation coefficient (for lesion counts per patient) and Cohen’s Kappa (for whether each patient had at least one enhancing lesion). A *p*-value <0.05 was considered significant.

## Results

A total of 318 MRI studies of independent patients were initially included. Of them, seven imaging studies were excluded owing to an incomplete imaging protocol and one imaging study owing to severe movement artifacts, impeding acceptable image review. Finally, 310 imaging studies were included in the analysis, which consisted of 216 women and 94 men, with a mean age of 46.7 years (standard deviation 12.2 years).

The first reader, the sub-specialized neuroradiologist, initially identified 117 contrast-enhancing foci on the T1WI+C images and 123 contrast-enhancing foci on the T1WI+C plus SWI+C images. The second reader, the 2nd-year radiology resident, identified 108 contrast-enhancing foci on the T1WI+C images and 121 definitive contrast-enhancing foci on the T1WI+C plus SWI+C images (Table 1). The Pearson correlation between the two readers was 0.982 for T1WI+C alone, and 0.994 for both sequences combined.

**Table 1:** Enhancing lesion count between the reading strategies for both readers (Reader 1 a board-certified neuroradiologist and Reader 2 a 2nd-year radiology resident). Significance between the reading strategies was evaluated for each reader using the paired Wilcoxon signed-rank test

	Reader 1		Reader 2	
Number of lesions	T1WI+C alone	Both modalities	T1WI+C alone	Both modalities
0	281	280	282	280
1	13	13	11	13
2	4	5	4	5
3	3	3	5	3
>4	9	9	7	7
Total lesions	117	123	108	121
*p*-value	0.0196		0.0364	

The expert reader identified 29 patients with enhancing lesions on the T1WI+C images; this number increased to 30 patients after T1WI+C plus SWI+C evaluation. The non-expert reader identified 28 patients with enhancing lesions on the T1WI+C images; this number increased to 30 patients after T1WI+C plus SWI+C evaluation (Table 2).

**Table 2:** Number of patients with any number of enhancing lesions between the reading strategies for both readers. Significance between the reading strategies was evaluated for each reader using the McNemar test. Cohen’s Kappa was calculated to determine the correlation between the readings of the two readers for each reading strategy

	T1WI+C alone	Both modalities	*p*-value
Reader 1	29	30	1
Reader 2	28	30	0.48
Cohen’s Kappa	0.981	1	–

We found no false-positive enhancement on the SWI+C images: All enhancing lesions on the SWI+C images, which were not identified on the T1WI+C images alone, demonstrated subtle enhancement on the second look with the T1WI+C images.

The increase in the total lesion count per patient was significant for both readers. In contrast, the increase in the presence of binary lesions was not significant for either of the two readers, as the main difference between the image review strategies was noted in the number of enhancing lesions identified in patients with several enhancing lesions.

**Fig. 1:**
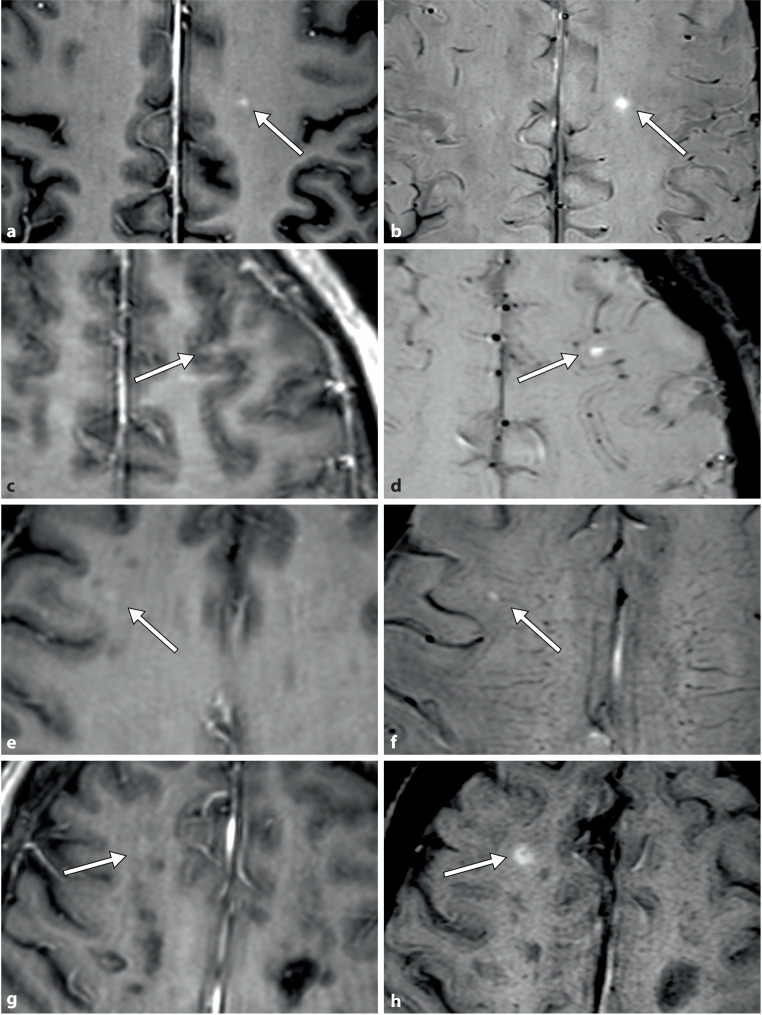
Examples of cases with improved enhancing lesion visualization with post-contrast susceptibility-weighted imaging (SWI+C). Each row corresponds to the same patient and MRI session, centered on the same lesion. The left column corresponds to contrast-enhanced T1-weighted imaging (T1WI+C), while the right column corresponds to SWI+C. The case in the top row was identified on T1WI+C but was more conspicuous and more straightforward to visualize on SWI+C. The remaining cases were initially missed on T1WI+C by one or both readers, but some subtle enhancements on T1WI+C became visible after identifying them on SWI+C

Examples of enhancing lesions that were more evident on the SWI+C images are presented in Fig. 1.

## Discussion

Our study explored the added value of performing SWI after endovenous contrast administration. In a homogeneous dataset of patients, we confirmed an increase in the number of detected enhancing lesions and the number of patients with enhancing lesions. Additionally, we found that the increase was greater for the less experienced reader and that the evaluation of contrast enhancement with SWI improved the agreement between the readers. Notably, on the second look with T1WI+C, subtle enhancement was identified in all cases in which it was initially observed on SWI+C alone and overlooked on T1WI+C alone (Fig. 1). This finding suggests that using SWI+C in combination with T1WI+C can enhance the reliability and consistency of detecting active lesions in patients with MS.

Enhancing lesions in patients with MS indicate active inflammation and serve as important markers of disease activity and dissemination, which is relevant for diagnosis, disease monitoring, and treatment optimization. A more accurate detection of these active lesions could allow for earlier or more aggressive treatment intervention, which could alter the disease course and improve the prognosis. Furthermore, detecting active enhancing lesions is critical in monitoring disease activity, assessing treatment response, and predicting disease progression, directly impacting disease management and patient outcomes. Therefore, a more sensitive and reliable method of detecting active enhancing lesions could significantly enhance the management and treatment of patients with MS.

The enhanced lesion detection with SWI+C could be attributed to the T1 shine-through effect [14]. Although SWI is primarily sensitive to T2* susceptibility (e.g., blood products, iron deposition), it also retains a T1 component. When the flip angle is close to or greater than the Ernst angle, the T1 relaxivity can dominate [14], leading to hyperintensities in tissues or lesions with significantly shortened T1 (e.g., subacute hematomas with methemoglobin [15] or gadolinium-bound lesions [10, 14]). Rather than representing a sporadic artifact, the T1 shine-through phenomenon is a predictable physical property of SWI that can reveal subtle gadolinium enhancement otherwise overlooked on standard T1WI+C. This concept has been successfully leveraged in neuro-oncology, wherein SWI+C improves the detection of hemorrhagic tumors and intratumoral vessels compared with T1WI+C [18, 19].

In line with our results, Do Amaral and colleagues previously highlighted the potential utility of SWI, particularly SWI+C, for enhancing active lesion detection in patients with MS [16]. They found that SWI+C was more sensitive to detecting active enhancing lesions than conventional T1WI+C.

Our findings align with and build upon the abovementioned findings. Our study replicates these results in a larger separate patient cohort and adds new insights regarding the applicability of SWI+C in the clinical context and its added value to the implications for clinical practice. Notably, we found a slight increase in the interobserver agreement between the expert and non-expert readers when SWI+C was used alongside T1WI+C, suggesting a potential role of this combined imaging approach in enhancing the consistency of lesion detection. This finding is relevant given the inherent variability in radiological interpretation, particularly in disease states such as MS, wherein accurate detection and quantification of active lesions can significantly impact clinical decision-making [3, 4]. Additionally, it is important to consider the influence of the timing between contrast administration and sequence acquisition on lesion detectability. Previous studies have indicated that a longer delay after contrast administration can lead to increased enhancement on T1-weighted images [20, 21]. In our study, SWI+C was performed before T1WI+C. Given the advantages of longer delays in enhancing lesion visibility, this sequence order could have placed SWI+C at a slight disadvantage. However, despite this potential limitation, the combined approach of SWI+C with T1WI+C improved lesion detection, underscoring its clinical relevance.

Although we acknowledge the growing interest in non-contrast imaging strategies such as DWI or advanced quantitative MRI, T1WI+C remains the clinical standard in most centers for detecting active MS lesions. Our findings do not preclude exploring these alternative sequences in future work.

The broader literature on SWI among patients with MS has predominantly focused on its potential diagnostic and prognostic implications owing to its sensitivity to iron deposition and ability to detect unique signs, such as the presence of central veins or the iron-rim sign [11, 12]. However, emerging evidence, as supported by our findings, indicates that SWI may have additional utility in monitoring disease activity through enhancing lesion detection.

Finally, our study further underscores the importance of MRI protocol standardization, an issue currently prevalent in MS imaging research. This has been highlighted in previous studies and reviews as a significant challenge and source of variability in research findings [10]. By demonstrating the potential added value of SWI+C in enhancing lesion detection, our findings contribute to the growing body of evidence supporting the need for standardized, comprehensive MRI protocols for patients with MS.

Despite the promising results, this study has limitations that should be acknowledged: First, this study adopted a retrospective design and was performed in a single institution, which may limit the generalizability of the findings. Second, SWI is a non-standardized imaging modality with marked inter-vendor variability [10]; this aspect should be considered before replicating the investigation in a different context. Third, we did not blindly compare between the sequences, which might have introduced subjectivity into the assessment. However, our aim was to analyze the feasibility and utility of SWI+C in the clinical setting, in which case it should accompany T1WI+C as indicated by current clinical guidelines. Lastly, owing to the retrospective nature of this study and the limited clinical data available, we could neither examine the clinical significance of the additionally detected lesions using SWI+C nor determine the impact of this improved detection on treatment decisions and patient outcomes.

Our study demonstrates that adding SWI+C to the standard MRI protocol can slightly improve the detection of active enhancing lesions in patients with MS, especially for less experienced readers. Furthermore, the combined use of T1WI+C and SWI+C appears to increase the interobserver agreement among radiologists, enhancing the reliability of detecting such lesions. This finding could inform clinical decisions and enhance the monitoring of disease activity among patients with MS. However, further prospective studies, possibly multicentric and involving clinical outcome data and a more comprehensive range of scanners and readers with different experience levels, are needed to validate the findings and explore their implications in the clinical context.

## Abbreviations

MS: Multiple sclerosis
SWI: Susceptibility-weighted imaging
SWI+C: Post-contrast susceptibility-weighted imaging
T1WI+C: Contrast-enhanced T1-weighted imaging
